# Low Vital Capacity and Electrocardiographic ST-T Abnormalities in Asymptomatic Adults

**DOI:** 10.1155/2012/460398

**Published:** 2012-05-22

**Authors:** Kei Nakajima, Yulan Li, Hiroshi Fuchigami, Hiromi Munakata

**Affiliations:** ^1^Division of Clinical Nutrition, Department of Medical Dietetics, Faculty of Pharmaceutical Sciences, Josai University, 1-1 Keyakidai, Sakado, Saitama 350-0295, Japan; ^2^Department of Internal Medicine, Social Insurance Omiya General Hospital, 453 Bonsai, Kita, Saitama 331-0805, Japan; ^3^Department of Health Care Center, Social Insurance Omiya General Hospital, 453 Bonsai, Kita, Saitama 331-0805, Japan

## Abstract

Studies have shown that low forced vital capacity (LFVC) is associated with atherosclerosis. However, it is unclear whether LFVC is associated with resting electrocardiographic ST-T abnormalities, a common finding that is prognostic for cardiovascular events. Therefore, pulmonary functions, ST-T abnormalities defined with Minnesota Code, and cardiometabolic risk factors were examined in a cross-sectional study of 1,653 asymptomatic adults without past history of coronary heart diseases. The prevalence of diabetes, metabolic syndrome, and ST-T abnormalities significantly increased with decreasing percent of predicted forced vital capacity (%PFVC). ST-T abnormalities were observed in 73 subjects (4.4% in total). Multiple logistic regression analysis showed that, compared with the highest quartile of %PFVC (≥99.7%), the lowest quartile of %PFVC (≤84.2%) was persistently associated with ST-T abnormalities even after further adjustment for diabetes or metabolic syndrome (odds ratio (95%CI): 2.44 (1.16–5.14) and 2.42 (1.15–5.10), resp.). Similar trends were observed when subjects were divided into quartiles according to percent of predicted forced expiratory volume in 1 second (FEV_1_), but not the ratio of FEV_1_/FVC. In conclusion, LFVC may be associated with ST-T abnormalities independent of metabolic abnormalities in asymptomatic adults, suggesting a plausible link between impaired pulmonary defects and cardiovascular diseases.

## 1. Introduction

Accumulating evidence has shown that not only impaired obstructive pulmonary function but also low forced vital capacity (LFVC) and impaired restrictive pulmonary function are associated with increased risk of mortality, partially owing to cardiovascular disease [1, 2]. Some studies have shown that LFVC is associated with arterial stiffness assessed by pulse-wave velocity [3, 4] and coronary artery calcification [5], which may reflect systemic atherosclerosis. In addition, LFVC and a restrictive pattern have been associated with critical metabolic abnormalities such as type 2 diabetes and metabolic syndrome (MetS) [2, 5–8], which are often followed by the development of cardiovascular disease [9, 10]. To date, however, the association between and the underlying mechanism of such pulmonary function defects and subclinical cardiovascular disease have been poorly understood. 

Meanwhile, ST depression, T-wave abnormalities, or both (ST-T abnormalities), particularly minor ST-T abnormalities, on the resting electrocardiogram are commonly observed in the general population without coronary heart disease [11–15]. Major ST-T abnormalities are an independent predictor for stroke [13], cardiovascular events, and increased mortality risk in people without heart disease at baseline [11, 12]. Likewise, many studies have provided evidence that even minor ST-T abnormalities are associated with increased risk for future cardiovascular disease independent of traditional risk factors [14, 16, 17], although the prognostic significance of minor ST-T abnormalities has not been well established [14].

In the light of this evidence, we hypothesized that impaired pulmonary functions, especially LFVC, may be associated with ST-T abnormalities irrespective of cardiometabolic risk factors, diabetes, and MetS. So far, no large epidemiological studies have examined the associations between impaired lung functions and ST-T abnormalities in asymptomatic individuals without overt heart disease.

Therefore, we examined lung functions, ST-T abnormalities that comprised major and minor ST-T abnormalities, cardiometabolic risk factors, diabetes, and MetS in a cross-sectional study of asymptomatic adults who underwent an annual checkup. Abnormal pulmonary function was evaluated by continuous pulmonary function variables including the percentage of predicted forced vital capacity (%PFVC).

## 2. Methods

The current study represents a series of studies performed in collaboration with Josai University, Sakado, Japan, and Social Insurance Omiya General Hospital, Saitama, Japan, that were conducted to explore the causalities and mechanism in lifestyle-related diseases. The study design and protocol have been described elsewhere [8]. The protocol was approved by the Ethical Committee of Josai University and the Council of the Hospital, and informed consent was obtained from all participants.

## 3. Subjects

**Figure 1:**
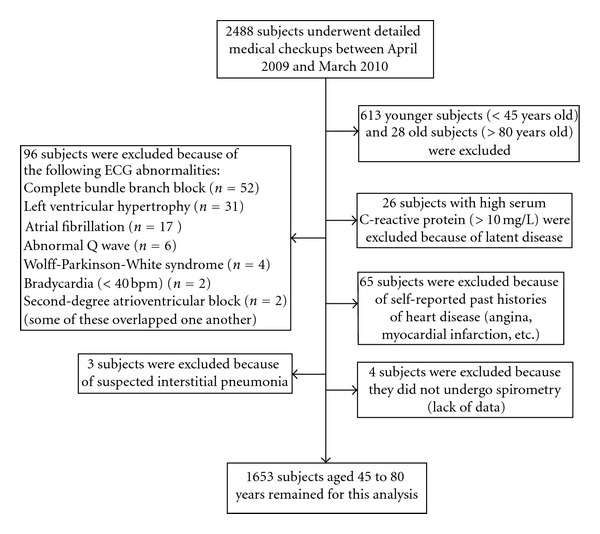
Exclusion criteria of subjects and flow chart.

Asymptomatic healthy subjects (*n* = 2,488) were randomly recruited from those who underwent complete medical checkups at their own will at the Health Care Center attached to Omiya General Hospital between April 2009 and March 2010. Most lived or worked close to the hospital (a small portion was treated in the hospital). Individuals who required immediate treatment for suspected cancers, pneumothorax, or infectious pneumonia, including tuberculosis, were not included from the beginning. All recruited subjects, who were free from overt disability and hemiplegia, completed a questionnaire about their lifestyle characteristics. Exclusion criteria of subjects and a flow chart are shown in Figure 1. Since people less than 45 years old are less likely to have ST-T abnormalities, first, subjects outside the age limitation of 45 to 80 years were excluded. Second, subjects with a high C-reactive protein (CRP) level (>10 mg/L) were excluded because of latent critical diseases or inflammation. Third, subjects with abnormal electrocardiograms, with the exception of ST-T abnormalities, were excluded. Subjects with a self-reported medical history of overt coronary heart disease were also excluded. Finally, after additional exclusion of subjects with suspected interstitial pneumonia based on chest X-ray findings and those without available spirometry data, 1,653 subjects (1,014 men and 639 women) were eligible for this analysis.

## 4. Laboratory Measurements and Determination of MetS and Diabetes

Anthropometric measurements and laboratory tests were carried out after an overnight fast. Waist circumference was measured at the height of the navel. Clinical and biochemical variables were measured automatically with standard methods using an autoanalyzer (Hitachi, Tokyo, Japan). The diagnosis of MetS was based on the modified Third Report of the National Cholesterol Education Program Expert Panel/Adult Treatment Panel criteria [9] with the following cutoff values: (1) systolic blood pressure of ≥130 mmHg and diastolic blood pressure of ≥85 mmHg; (2) triglycerides of ≥150 mg/dL; (3) low high-density lipoprotein of <40 mg/dL for men and <50 mg/dL for women; (4) fasting plasma glucose of ≥100 mg/dL; (5) waist circumference of ≥90 cm for men and ≥80 cm for women. We took ethnic-specific values for waist circumference into consideration. MetS was diagnosed in subjects fulfilling three or more of the above five criteria. Subjects receiving medication for any of these components were defined as having the component. Diabetes was defined as a fasting plasma glucose of ≥126 mg/dL or HbA1c of ≥6.5% according to the American Diabetes Association criteria [18], or treatment with oral hypoglycemic drugs or insulin. HbA1c was converted to National Glycohemoglobin Standardization Program (NGSP) levels by the formula HbA1c (%) (NGSP) = HbA1c (JDS) (%) + 0.4% [19].

## 5. Spirometry

Pulmonary function tests were performed with a spirometry analyzer (Autospiro-507, Minato Medical Science Co., Ltd., Osaka, Japan). The test was performed by trained technicians with the subject in a standing position. The %PFVC, percentage of predicted forced expiratory volume in 1 second (%PFEV_1_), and ratio of forced expiratory volume in 1 second to observed forced vital capacity (FEV_1_/FVC) were calculated as in our previous report [8]. We quoted the standard predicted values for FVC, FEV_1_, and FEV_1_/FVC from data published by the Japanese Respiratory Society in 2001 [20].

## 6. Electrocardiogram

Standard 12-lead electrocardiograms were recorded with an electrocardiogram recorder (Cardio Base FDX-4521, Fukuda Denshi Co., Ltd., Tokyo, Japan). ST-T abnormalities were defined with the Minnesota Code [21]. Minor ST-T abnormalities included the following: ST junction depression of <0.5 mm (MC 4-3); ST junction depression of >1 mm and ST segment ascent, that is, upslope (MC 4-4); T wave flat, diphasic, or inverted by <1 mm (MC 5-3). Major ST-T abnormalities included (MC 4-1), (MC 4-2), (MC 5-1), and (MC 5-2). The findings were first determined by experienced physicians and subsequently confirmed by trained medical laboratory technicians, all of whom exclusively belong to the Social Insurance Omiya General Hospital and were blinded to individuals' data. The minor and major ST-T abnormalities were combined and analyzed together in this study because, on the checkup sheets, minor and major ST abnormalities were recorded together indistinguishably.

## 7. Statistical Analysis

Data are expressed as means ± SD or median/geometric mean (interquartile range). Subjects were divided into quartiles according to continuous pulmonary function variables: %PFVC, %PFEV_1_, and FEV_1_/FVC. *P* values for continuous variables and categorical variables were determined with ANOVA and the *χ* ^2^-test, respectively.

Multivariate logistic regression models were used to examine the associations between the lowest quartiles (Q1) of lung functions and ST-T abnormalities compared with the corresponding highest quartiles (Q4), controlling for clinical confounding factors including diabetes and MetS. This analysis yielded odds ratios (OR) and 95% confidential intervals (95% CI). Tests for linear trends (*P* for trend) were calculated by treating quartile categories (Q1–Q4) as a continuous variable (i.e., 1–4), and the same model analysis was conducted. In this study, hypertension (≥130/85 mmHg) was considered as a special confounder that substantially interferes with the associations because hypertension is likely to affect ST-T abnormalities and the decline of FVC [13, 22]. Statistical analyses were performed using SPSS software version 18.0 (SPSS-IBM, Chicago, IL) and Statview version 5.0 (SAS Institute, Cary, NC). Values of *P* < 0.05 were considered to be statistically significant.

## 8. Results

Overall, most subjects in this study had relatively good profiles in terms of anthropometric and biochemical parameters, including pulmonary functions tests (Table 1). The prevalence of cardiometabolic risk factors, diabetes, MetS, and ST-T abnormalities significantly increased with decreasing %PFVC (toward Q4).

Multiple logistic analysis showed that compared with the highest quartile of %PFVC (≥99.7%) (Q1), the lowest quartile (≤84.2%) (Q4) was significantly associated with ST-T abnormalities (Table 2). This association remained significant even after adjustment for age, sex, smoking, alcohol consumption, frequency of exercise, self-reported past history of stroke, waist circumference, and CRP (both as a continuous variable), with significant *P* for trends. Moreover, extended adjustment for diabetes (Model 4) or MetS (Model 5) attenuated but did not remarkably alter the associations. In these conditions, diabetes and MetS were significantly associated with ST-T abnormalities (OR (95% CI), 2.18 (1.18–4.03), *P* = 0.01 and 2.20 (1.23–3.93), *P* = 0.008, resp.; data not shown).

Likewise, similar trends were observed when subjects were divided into quartiles according to %PFEV_1_. However, observed associations between lowest quartile of %PFEV_1_ and ST-T abnormalities were not significant after adjustment for diabetes or MetS. In contrast, no significant associations between lowest quartile of FEV_1_/FVC and ST-T abnormalities were observed in comparison with the highest quartile of FEV_1_/FVC, irrespective of adjustments for confounders.

Meanwhile, the lowest quartile of %PFVC was significantly associated with hypertension (≥130/85 mmHg) (OR (95%CI), 1.76 (1.30–2.38), *P* = 0.0003, data not shown) compared with the highest quartile, even after full adjustments for confounders in Model 3 of Table 2. However, the significant association between LFVC and ST-T abnormalities persisted after further adjustments for confounders in Model 3 of Table 2, plus hypertension or medication for hypertension (OR (95%CI), 2.43 (1.15–5.11), *P* = 0.02 and 2.58 (1.23–5.40), *P* = 0.01, resp., data not shown).

## 9. Discussion

This study was conducted to investigate the relationship of impaired lung functions with resting electrocardiographic ST-T abnormalities in a cross-sectional analysis of asymptomatic adults without past history of heart disease. We found that LFVC and probably low FEV_1_, but not low FEV_1_/FVC, were significantly associated with ST-T abnormalities, which was attenuated by further adjustment for cardiometabolic risk factors, diabetes (presumably mostly type 2 diabetes in this study), or MetS, but remained significant. Notably, cardiometabolic risk factors included circulating CRP and waist circumference, surrogate markers of systemic inflammation, and amount of abdominal fat, respectively. Collectively, the current findings suggest that LFVC, which often reflects restrictive pulmonary function pattern, may be independently associated with ST-T abnormalities irrespective of clinical confounders, diabetes, and MetS, whereas obstructive pulmonary function defects are not.

So far, no clinical study has examined the relationship of LFVC with electrocardiographic abnormalities with the exception of a study by Sideris and Katsadoros [23]. This study reported a negative correlation between vital capacity and number of abnormalities, such as a rightward shift of the P-wave and clockwise rotation of QRS, but it did not address ST or T-wave abnormalities. Thus, to our knowledge, this study is the first to demonstrate the association between LFVC and ST-T abnormalities, which is conceivably prognostic for cardiovascular events in asymptomatic people [14, 16, 17].

According to previous studies, LFVC and restrictive pulmonary defects have been associated with coronary artery calcification [5] and arterial stiffness [3, 4]. Because artery calcification and arterial stiffness generally reflect atherosclerosis and ischemic cardiovascular diseases, the current results are consistent with these previous studies. Generally, ST-T abnormalities are observed in various conditions, such as ischemia, hypokalemia, cardiomyopathy, and pulmonary embolism [15]. Ohira et al. [13] mentioned that minor ST-T abnormalities may reflect an end-organ effect of long-term hypertension because hypertensive men with minor ST-T abnormalities tended to have longer durations of hypertension in their study. Indeed, arterial stiffness assessed with pulse-wave velocity is substantially affected by hypertension [24]. Furthermore, a recent study showed that decline in FVC predicted incident hypertension in young apparently healthy individuals [22]. Actually, in our study, LFVC was robustly associated with hypertension, even after full adjustments for critical confounders. Nevertheless, the significant association between LFVC and ST-T abnormalities persisted after further adjustments for confounders plus hypertension or medication for hypertension. Therefore, although hypertension likely contributes in part to the observed associations through the close interrelationship of lung with cardiovascular system, other unknown factors may principally interfere with the associations between LFVC and ST-T abnormalities.

Considering that LFVC was associated with ST-T abnormalities independently of cardiometabolic factors, circulating CRP, diabetes, and MetS, which are proatherosclerotic and proinflammatory [9, 10], several factors not examined in this study, such as insulin resistance, oxidative stress, or subclinical hypoxia, might interfere with the relationship between LFVC and ST-T abnormalities. In accordance with this, previous studies have hypothesized that insulin resistance is a fundamental element for the pathophysiology of LFVC and restrictive lung function [2, 6, 7].

Meanwhile, many studies in the past decade have shown that a predisposition for cardiovascular disease and impaired pulmonary functions (low vital capacity and low FEV_1_) are associated with low birth weight [25, 26], possibly via physiological alterations such as increased adrenocortical and sympathoadrenal responses to an adverse fetal environment [27]. LFVC and ST-T abnormalities might then relate to such potential factors as epiphenomena.

Alternatively, physicochemical factors might interfere with the associations. Of note, the American Heart Association recently updated its scientific statement to describe that exposure to particulate-matter air pollution contributes to cardiovascular morbidity and mortality [28]. It highlighted several possible biological mechanisms secondary to pulmonary oxidative stress, inflammation, and an impaired lung autonomic nervous system that might result in vascular dysfunction and ST-segment depression irrespective of metabolic abnormalities.

Several limitations should be mentioned. First, because of the nature of cross-sectional studies, causality remains unknown and must be elucidated in large prospective studies. Second, it was not possible to distinguish between major and minor ST-T abnormalities in this study because the electrocardiogram findings on individual checkup sheets in combinations of major and minor ST-T abnormalities were recorded together indistinguishably. However, most ST-T abnormalities were likely to be minor ST-T abnormalities because the prevalence of minor ST-T abnormalities is approximately 2-fold greater than that of major ST-T abnormalities in the Japanese population [13].

Third, all subjects in this study were instructed by staff members trained in spirometry, and the subjects performed a few rehearsals. Nevertheless, it was not clear whether all subjects had acceptable results in the actual test, especially the elderly, regardless of cognitive status, physical performance, or education level. This is likely to result in potential bias of the outcomes.

Finally, most subjects were healthy with good profiles in terms of various parameters, although a small portion of them had several critical conditions such as diabetes or history of stroke. Therefore, the current findings may not be applicable to other populations that have more cardiometabolic risk factors and ST-T abnormalities, where the observed associations, if any, might be dependent on hypertension, diabetes, or metabolic syndrome.

## 10. Conclusion

LFVC may be associated with ST-T abnormalities independently of cardiometabolic risk factors, diabetes, and MetS in asymptomatic adults without overt heart disease. Our results suggest that there is a mechanism linking low vital capacity with common electrocardiographic abnormalities and that this mechanism is prognostic for increased risk for cardiovascular diseases, which needs to be confirmed in further larger studies.

## Figures and Tables

**Table 1:** Clinical characteristics of subjects according to %PFVC quartiles.

	Total	Q1 (highest)	Q2	Q3	Q4 (lowest)	*P* values
*n*	1,653	412	413	414	414	
Men, *n* (%)	1,014 (61.3)	205 (49.8)	248 (60.0)	273 (65.9)	288 (69.6)	<0.0001
Age, y	58.2 ± 8.6	58.6 ± 8.6	57.3 ± 8.3	58.3 ± 8.5	58.6 ± 8.9	0.12
BMI, kg/m^2^	23.3 ± 3.1	22.8 ± 2.6	23.1 ± 3.0	23.3 ± 3.0	23.9 ± 3.1	<0.0001
Waist circumference, cm	82.3 ± 8.5	80.5 ± 7.5	81.8 ± 8.56	82.6 ± 8.3	84.3 ± 9.2	<0.0001
Systolic blood pressure, mmHg	123 ± 19.0	120 ± 17.9	123 ± 18.1	124 ± 19.6	127 ± 19.9	<0.0001
Diastolic blood pressure, mmHg	76.9 ± 12.6	74.3 ± 12.2	77.4 ± 12.1	77.5 ± 12.7	78.6 ± 12.8	<0.0001
Triglyceride, mg/dL	96 (69–138)	84 (63–120)	90 (66–133)	103 (74–142)	107 (74–155)	<0.0001
HDL-C, mg/dL	62.0 ± 15.3	64.8 ± 15.1	62.2 ± 15.3	61.5 ± 15.5	59.4 ± 15.1	<0.0001
Fasting plasma glucose, mg/dL	102 ± 18.5	99.8 ± 16.2	101±16.2	103 ± 18.6	106 ± 22.1	<0.0001
HbA1c, % (NGSP)	5.4 ± 0.6	5.3 ± 0.6	5.3 ± 0.5	5.4 ± 0.6	5.5 ± 0.7	<0.0001
CRP, mg/L	0.51 (0.30–0.80)	0.44 (0.30–0.60)	0.49 (0.30–0.70)	0.54 (0.30–0.90)	0.60 (0.30–1.00)	<0.0001
FVC, mL	3, 133 ± 763	3, 500 ± 834	3,274±729	3, 046 ± 661	2713 ± 574	<0.0001
FEV_1_, mL	2, 496 ± 618	2, 761 ± 645	2,605±593	2, 442 ± 559	2, 176 ± 513	<0.0001
%PFVC (range)	92.3 (55.6–138)	108 (99.7–138)	95.5 (91.9–99.6)	88.0 (84.3–91.8)	78.4 (55.6–84.2)	—
%PFEV_1_	89.1 ± 13.4	103 ± 10.3	91.9 ± 7.7	85.3 ± 7.3	76.0 ± 9.9	<0.0001
FEV_1_/FVC ratio, %	79.9 ± 6.8	79.2 ± 5.7	79.8 ± 6.4	80.3 ± 6.6	80.2 ± 8.1	0.11
ST-T abnormalities, *n* (%)	73 (4.4)	11 (2.7)	13 (3.1)	18 (4.3)	31 (7.5)	0.003
Metabolic syndrome, *n* (%)	298 (18.0)	51 (12.4)	58 (14.0)	80 (19.3)	109 (26.3)	<0.0001
Components of metabolic syndrome						
Elevated waist circumference, *n* (%)	515 (31.2)	111 (26.9)	119 (28.8)	129 (31.2)	156 (37.7)	0.006
Elevated blood pressures, *n* (%)	690 (41.7)	134 (32.5)	163 (39.5)	186 (44.9)	207 (50.5)	<0.0001
Elevated triglyceride, *n* (%)	342 (20.7)	61 (14.8)	78 (18.9)	94 (22.7)	109 (26.3)	0.0003
Low HDL-C, *n* (%)	99 (6.0)	22 (5.3)	22 (5.3)	19 (4.6)	36 (8.7)	0.06
Elevated fasting plasma glucose, *n* (%)	749 (45.3)	152 (36.9)	182 (44.0)	189 (45.7)	226 (54.6)	<0.0001
Diabetes, *n* (%)	158 (9.6)	27 (6.5)	35 (8.5)	37 (8.9)	59 (14.3)	0.001
Medical history of						
Stroke, *n* (%)	29 (1.8)	9 (2.2)	2 (0.5)	7 (1.7)	11 (2.7)	0.10
Medications for						
Hypertension, *n* (%)	322 (19.5)	60 (14.6)	78 (18.9)	93 (22.5)	91 (22.0)	0.02
Hypercholesterolemia, *n* (%)	202 (12.2)	48 (11.7)	43 (10.4)	58 (14.0)	53 (12.8)	0.43
Diabetes, *n* (%)	63 (3.6)	10 (2.4)	13 (3.1)	12 (2.9)	24 (5.8)	0.04
Current smoker, *n* (%)	382 (23.1)	94 (22.8)	86 (20.8)	92 (22.2)	110 (26.6)	0.24
Daily alcohol consumption, *n* (%)	547 (33.1)	143 (34.7)	133 (32.2)	144 (34.9)	127 (30.7)	0.51
Regular exercise, *n* (%)	599 (36.3)	155 (37.6)	144 (34.9)	161 (38.9)	139 (33.6)	0.37

Data are means ± SD. Triglyceride and CRP are expressed as medians and geometric means with interquartile range, respectively. %PFVC is expressed as means (range).

Regular exercise: ≥30 min exercise per session ≥2 days/week. Elevated waist circumference: ≥90 cm for men and ≥80 cm for women. Elevated blood pressures: ≥130/85 mmHg. Elevated triglyceride: ≥150 mg/dL. Low HDL-C: <40 mg/dL for men and <50 mg/dL for women. Elevated fasting plasma glucose: ≥100 mg/dL.

^a^Proportion of T-wave abnormalities of all ST-T abnormalities in each quartile.

*P* values for continuous variables and categorical variables were determined with ANOVA and the *χ* ^2^-test, respectively.

BMI: body mass index; HDL-C: high-density lipoprotein cholesterol; CRP: C-reactive protein; FVC: forced vital capacity; FEV_1_: forced expiratory volume in 1 second; %PFVC: percentage of predicted forced vital capacity; %PFEV_1_: percentage of predicted forced expiratory volume in 1 second; FEV_1_/FVC: ratio of forced expiratory volume in 1 second to observed forced vital capacity; NGSP: National Glycohemoglobin Standardization Program.

**Table 2:** Odds ratio (95% CI) of lung function quartiles for ST-T abnormalities.

	Q1 (highest)	Q2	Q3	Q4 (lowest)	*P* for trend*
%PFVC, *n*	412	413	414	414	
Model 1	1	1.19 (0.52–2.68)	1.66 (0.77–3.55)	2.95 (1.46–5.95)^†^	0.0007
Model 2	1	1.33 (0.59–3.03)	1.83 (0.85–3.95)	3.27 (1.60–6.69)^†^	0.0004
Model 3	1	1.30 (0.56–3.00)	1.74 (0.79–3.81)	2.59 (1.23–5.42)^ §^	0.006
Model 4	1	1.29 (0.56–2.97)	1.74 (0.79–3.83)	2.44 (1.16–5.14)^§^	0.009
Model 5	1	1.29 (0.56–3.00)	1.69 (0.77–3.73)	2.42 (1.15–5.10)^§^	0.01

%PFEV_1_, *n*	412	415	414	412	
Model 1	1	0.67 (0.31–1.47)	1.06 (0.53–2.13)	1.87 (1.002–3.51)^§^	0.02
Model 2	1	0.78 (0.36–1.72)	1.35 (0.66–2.77)	2.42 (1.23–4.75)^ §^	0.003
Model 3	1	0.76 (0.34–1.69)	1.16 (0.56–2.42)	2.09 (1.04–4.21)^§^	0.02
Model 4	1	0.75 (0.34–1.69)	1.10 (0.53–2.31)	2.00 (0.99–4.0.4)	0.02
Model 5	1	0.76 (0.34–1.70)	1.13 (0.54–2.35)	2.00 (0.99–4.06)	0.02

FEV_1_/FVC, *n*	412	413	416	412	
Model 1	1	1.41 (0.76–2.63)	0.71 (0.34–1.46)	0.94 (0.48–1.86)	0.42
Model 2	1	1.38 (0.74–2.59)	0.60 (0.28–1.26)	0.74 (0.36–1.56)	0.15
Model 3	1	1.56 (0.82–2.98)	0.69 (0.32–1.48)	0.94 (0.44–1.98)	0.41
Model 4	1	1.52 (0.80–2.91)	0.69 (0.32–1.49)	0.94 (0.44–1.98)	0.42
Model 5	1	1.55 (0.81–2.97)	0.69 (0.32–1.50)	0.93 (0.44–1.97)	0.41

Data are expressed as odds ratio (95% confidence interval) with references of highest quartiles (Q1).

Model 1: unadjusted; Model 2: adjusted for age, sex, height, and current smoking (versus nonsmokers); Model 3: Model 2 plus adjustment for daily alcohol consumption (versus infrequent/no alcohol consumption), regular exercise (versus no regular exercise), waist circumference, log-transformed CRP, and self-reported past history of stroke; Model 4: Model 3 plus adjustment for diabetes; Model 5: Model 3 plus adjustment for MetS.

*P values correspond to tests for linear trends across quartile treated as a continuous value.

^†^ *P* < 0.005, ^§^ *P* < 0.05 for each association.
